# Using Glycated Albumin and Stimulated C-Peptide to Define Partial Remission in Type 1 Diabetes

**DOI:** 10.3389/fendo.2022.938059

**Published:** 2022-07-19

**Authors:** Mei Shi, Xiaolin Ji, Yuting Xie, Ting Zhong, Rong Tang, Li Fan, Xia Li

**Affiliations:** National Clinical Research Center for Metabolic Diseases, Key Laboratory of Diabetes Immunology (Central South University), Ministry of Education, and Department of Metabolism and Endocrinology, The Second Xiangya Hospital of Central South University, Changsha, China

**Keywords:** remission phase, type 1 diabetes, glycated albumin, C-peptide, insulin resistance

## Abstract

**Objective:**

To propose a new definition of partial remission (PR) for patients with type 1 diabetes (T1D) of all-ages using insulin dose and glycated albumin (GA), and find the optimal cut-off values for stimulated C-peptide to diagnose PR in different age-groups.

**Research Design and Methods:**

Patients with newly diagnosed T1D (n=301) were included. GA/insulin dose was used to diagnose PR, and insulin dose-adjusted glycated albumin (IDAGA) was proposed to facilitate clinical application. The optimal diagnostic levels of IDAGA and stimulated C-peptide were determined in different age-groups (≤ 12y, 12-18y and ≥ 18y). Furthermore, the diagnostic consistency between different PR definitions was studied.

**Results:**

GA≤ 23%/insulin dose ≤ 0.5u/kg/day was used to define PR, and IDAGA (GA (%) + 40 * insulin dose(u/kg/day)) ≤ 40 was feasible in all age-groups. Whereas, the optimal diagnostic level showed difference for stimulated C-peptide (265.5, 449.3 and 241.1 pmol/L for the ≤ 12y, 12-18y and ≥ 18y age-group, respectively). About 40% of patients met the PR definition by stimulated C-peptide but not GA/insulin dose or IDAGA, who showed dyslipidemia and higher insulin resistance.

**Conclusions:**

A new definition of the PR phase is proposed using GA/insulin dose, and the calculated IDAGA≤ 40 applies to all age-groups. The stimulated C-peptide to diagnose PR is the highest in the 12-18y age-group, which reflects the effect of puberty on metabolism. For patients with insulin resistance, it is not recommended to use stimulated C-peptide alone to diagnose PR.

## Introduction

Type 1 diabetes (T1D) is a chronic disease characterized by persistent autoimmune destruction of β cells, leading to lifelong dependence on exogenous insulin (1). During the progression of this disease, some of the newly diagnosed patients might experience a special period termed as the partial remission (PR) phase, which is characterized by satisfactory glycemic control with reduced daily insulin requirements and the transient restoration of β cell function (2). The improved glycemic control during the PR calls for an adjustment of the insulin regiment in order to avoid acute events, and the presence of PR phase is associated with a lower risk of developing chronic complications (3–5). Therefore, it is of great clinical and research significance to guarantee the diagnostic accuracy of PR.

The following are the three definitions of PR frequently applied in clinical practice (2): 1) low dose of insulin requirement (< 0.3 or 0.5 u/kg/day) and satisfactory glycemic control (HbA1c < 7.0 or 7.5%); 2) a calculated insulin dose‐adjusted A1c (IDAA1C) index ≤9 (IDAA1C = HbA1c(%) + [4×insulin dose (u/kg/day)]); 3) stimulated C-peptide ≥300pmol/L. Since insulin requirement and HbA1c are easy to obtain, the first two definitions are more commonly used in the clinic. However, HbA1c reflects glycemic changes over the past 2-3 months, and is therefore lagged when used to diagnose PR. Whereas, glycated albumin (GA) reflects fluctuations in blood glucose within the previous 2-3 weeks due to the shorter half-life of albumin (6), so it would be a more sensitive measurement to use. In addition, T1D shows age-related heterogeneity across a broad range of clinical features, including HbA1c, insulin requirement and C-peptide (7–9), so personalized diagnostic criteria of PR for different age-groups are needed.

Therefore, our study aims to propose a new definition of the PR phase using GA and insulin dose, and verify whether the diagnostic criteria of PR by IDAGA and stimulated C-peptide are suitable for different age-groups.

## Research Design and Methods

### Subjects and Designs

**Figure 1:**
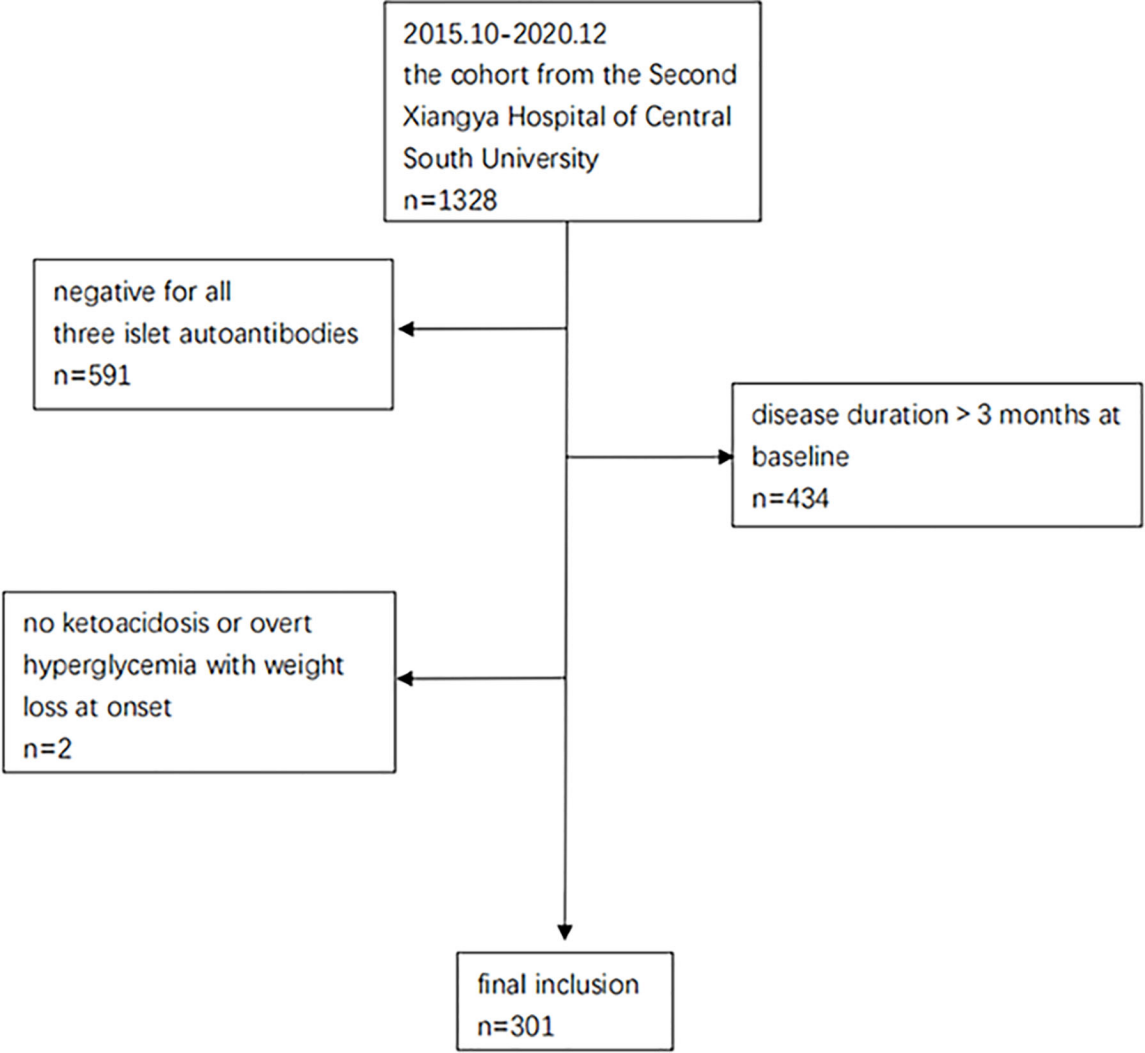
Enrollment flow chart of the study.

From October 2015 to December 2020, 1328 patients from the T1D cohort at the Second Xiangya Hospital of Central South University were screened (Clinical Trials.gov ID: NCT03610984). This cohort has been described in detail in previous studies (10, 11). Participants who met the following criteria were included for this study: 1) diagnosis of diabetes based on criteria from the World Health Organization (1999) (12), 2) ketoacidosis or overt hyperglycemia with weight loss thus starting insulin treatment from the time of disease onset, 3) positivity for at least 1 of the 3 islet autoantibodies measured (glutamic acid decarboxylase antibody [GADA], insulinoma associated protein 2 antibody [IA-2A], or zinc transporter antibody [ZnT8A]).4) disease duration of no more than 3 months at baseline. Among all the 1328 patients, 591 cases were excluded because they had negative autoantibodies, 434 cases were excluded because of long disease durations (>3 months) at baseline, and 2 were excluded due to no ketoacidosis or overt hyperglycemia with weight loss at onset. Ultimately, a total of 301 patients were included for the current study (**Figure 1**), and their PR status was diagnosed retrospectively. All subjects participating in the dynamic cohort had signed informed consent. The study was approved by the Research Ethics Committee of Second Xiangya Hospital, Central South University (approval number: 2017-028) and conducted in accordance with the Declaration of Helsinki guidelines.

The baseline characteristics recorded include: gender, age of onset, duration of diabetes, diabetic ketosis/ketoacidosis at onset, waist circumference, blood pressure, hip circumference, daily insulin dosage (U/kg/day), HbA1c, glycated albumin (GA), serum lipid, fasting C-peptide (FCP), stimulated C-peptide, islet autoantibodies.

The patients were followed up at an interval of 3-6 months, and the clinical information collected at each follow-up includes: age, duration of diabetes, waist circumference, hip circumference, blood pressure, daily insulin dosage, HbA1c, GA, serum lipid, FCP, and stimulated C-peptide after mixed meal tolerance test (MMTT).

### Measurements of HbA1c, GA, Serum Lipid and Islet Autoantibodies

HbA1c was measured by automated liquid chromatography (VARIANT II Hemoglobin Testing System; Bio-Rad Laboratories, Hercules, CA). GA was tested by the enzymatic method using Lucica Glycated Albumin‐L (Asahi Kasei Pharma Corporation, Tokyo, Japan) as the measuring reagent and Mindray BS240 (Mindray medical International Limited, Shenzhen, China) as the measuring instrument. Total cholesterol (TC), triglyceride (TG), high density lipoprotein cholesterol (HDL-C) and low density lipoprotein cholesterol (LDL-C) were determined by a Hitachi 7170 analyzer (Boehringer Mannheim, Mannheim, Germany). GADA, IA-2A, and ZnT8A were detected by radioimmunoassay as previously reported (13). The cut-off points for GADA, IA-2A, and ZnT8A positivity were defined as the 99th percentile of 405 healthy control subjects. Suspected positive samples were tested twice for confirmation. The sensitivities and specificities of the current GADA, IA-2A, and ZnT8A were evaluated in the Islet Autoantibody Standardization Program (IASP 2012).

### MMTT for C-Peptide Testing

A standard 543.6 kcal MMTT, with 44.4% carbohydrates, 47.7% fat and 7.9% protein, were performed before 10:00 am after an overnight fast. Long-acting insulin was used normally on the day before the MMTT, but short-acting insulin after breakfast on the test day was withheld. The MMTT was conducted with the fasting glucose levels between 3.9 and 11.1 mmol/L (70 and 200 mg/dL) and at least two weeks after the correction of ketoacidosis. Serum C-peptide levels were assessed at 0 (fasting C-peptide, FCP) and 120 minutes (stimulated C-peptide) after the first bite of a mixed meal. C-peptide levels were measured by a chemiluminescence method using the Advia Centaur System kit (Siemens, Munich, Germany).

### Insulin Resistance

Insulin resistance was measured by the estimated glucose disposal rate (eGDR) based on the following equation: eGDR (mg/kg/min) = 24.31-(12.22×waist to hip ratio)-(3.29×hypertension, yes= 1, no= 0)-(0.57×HbA1c), where the hypertension status was defined as blood pressure higher than 140/90 mmHg (14).

### Statistical Analysis

This study was mainly a diagnostic test to explore the optimal cut-off point for defining the PR by receiver operator characteristic (ROC) curves. We calculated that a sample size of 62 (PR:31; non-PR:31) patients would provide a statistical power of 0.8 to find the optimal cut-off point for PR diagnosis (H0: AUC=0.5, H1: AUC=0.7) at an alpha level of 0.05 (two-sided) on the basis of a one-sample ROC curve (calculated by Power Analysis and Sample Size, PASS). After the enrollment process, 301 eligible patients were included, which fulfilled the calculated sample size.

Data are presented as the mean ± standard deviation (SD) or median (25th percentile-75th percentile), or according to the specific notes in the article. Spearman′s rank correlation and simple linear regression were used to determine the association between HbA1c and GA so as to find the cut-off value for GA to define PR. Logistic regression was used to calculate the regression coefficient of GA and insulin dose. When comparing the stimulated C-peptide levels of patients in PR and not in PR, multiple follow-up points for the same patient were included for analysis and generalized estimation equation (GEE) was performed for these repeated- measures data. When comparing clinical characteristics between patients who met the criteria of PR by stimulated C-peptide but not GA/insulin or IDAGA and by both stimulated C-peptide and GA/insulin or IDAGA, the follow-up points where patients first met the criteria above were included, and student’s t test was carried out for variables with the normal distribution, while Mann-Whitney U test was used for data with non-normal distribution. SPSS 25.0 was used for the analysis above. The optimal cut-off point was determined by receiver operator characteristic (ROC) curve and restricted cubic spline (RCS). ROC was employed to find the optimal cut-off point of stimulated C-peptide and IDAGA to define the PR in different age-groups, with the GA/insulin dose being the diagnostic criteria. In addition, RCS was used to verify these optimal cut-off points obtained from ROC. The analysis was done using R version 4.0.5. Statistical significance was considered at P < 0.05.

## Results

A total of 301 patients with new-onset T1D (median age: 13.2 years; male: 54.8%) were enrolled in our study, all of whom were positive for at least one islet autoantibodies (**Figure 1**). The patients were followed up for a median duration of 18.9 months (5.9-30.5months), and their PR status was diagnosed retrospectively. Baseline characteristics of the subjects enrolled in our study are presented in **Table 1**.

**Table 1:** Characteristics of study participants at baseline.

Clinical characters	T1D (n = 301)
Age (years)	13.2 (8.2-26.1)
Male (%)	165 (54.8%)
Age at onset (years)	13.1 (8.2-26.0)
Duration of diabetes (months)	1.2 (0.7-1.8)
DK/DKA at onset (%)	243 (91.0%)
Fasting C-peptide (pmol/L)	121.0 (56.3-171.4)
Stimulated C-peptide (pmol/L)	270.6 (150.8-462.6)
Hemoglobin A1c (%)	10.6 (8.5-12.7)
Insulin dose (u/kg/day)	0.6 (0.4-0.8)
Systolic blood pressure (mmHg)	107.0 (98.0-117.0)
Diastolic blood pressure (mmHg)	67.0 (59.0-73.8)
Waist to hip ratio (%)	84.8 (80.0-90.2)
HDL-C (mmol/l)	1.4 (1.1-1.7)
LDL-C (mmol/l)	2.3 (1.8-2.9)
Total Cholesterol (mmol/l)	4.3 (3.6-5.0)
Triglyceride (mmol/l)	0.8 (0.6-1.1)
eGDR (mg/kg/min)	8.0 (6.6-9.1)

Data are expressed as % (n), mean ± SD and median (25th-75th percentile). DK, diabetic ketosis; DKA, diabetic ketoacidosis, HDL-C: high density lipoprotein cholesterol; LDL-C: low density lipoprotein cholesterol; eGDR: estimated glucose disposal rate.

### Definition of PR by GA in Combination With Insulin Requirement

GA was positively correlated with HbA1c (r=0.7; R²=0.4; GA=2.6*HbA1c+3.9), with GA of 23.4% corresponding to HbA1c of 7.5%. As HbA1c ≤ 7.5% in combination with an insulin requirement of ≤0.5u/kg/day was commonly used to define the PR phase, PR was defined as GA ≤ 23.0% and an insulin requirement of ≤ 0.5u/kg/day in our study.

### Definition of PR by Insulin Dose-Adjusted Glycated Albumin

**Figure 2:**
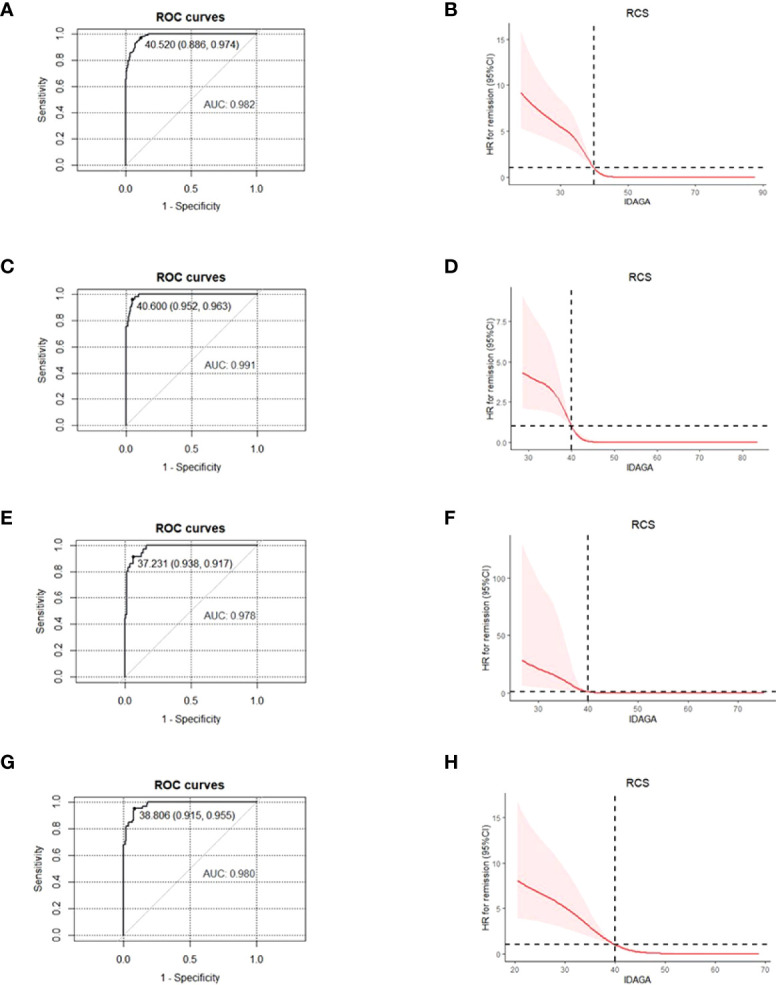
For patients of different age- groups, ROC and RCS curves were used to find the optimal cut-off point for IDAGA for IDAGA to define the partial remission, with GA≤ 23% and insulin dose ≤0.5 u/kg/day being the diagnostic criteria. All age- groups: ROC **(A)**, RCS **(B)**; ≤ 12 years group: ROC **(C)**, RCS **(D)**; 12-18 years group, ROC **(E)**, RCS **(F)**; ≥ 18 years group, ROC **(G)**, RCS **(H)**. ROC, receiver operating characteristic; RCS, restricted cubic spline; IDAGA, insulin dose-adjusted glycated albumin; GA, glycated albumin.

In order to make the new PR definition practical to use in the clinic, logistic regression was performed to transform the definition into a quantitative measure. During the analysis, GA and insulin dose were taken as independent variables, and the PR diagnosis was the dependent variable. The regression coefficient was -20.5 for insulin dose and -0.5 for GA. As the ratio was roughly 40 between the two independent variables, IDAGA = GA (%) + 40 * insulin dose (u/kg/day) was proposed. With GA/insulin dose being the diagnostic criteria, ROC and RCS curves showed that the optimal cut-off point for IDAGA was 40, which means a calculated IDAGA ≤ 40 could be used to define PR (**Figures 2A** **, B**). Further analysis showed that IDAGA ≤ 40 was suitable in all age-groups (**Figure 2**).

### Stimulated C-Peptide Levels During PR and the Age-Dependent Cut-Off Values for Stimulated C-Peptide to Define PR

**Figure 3:**
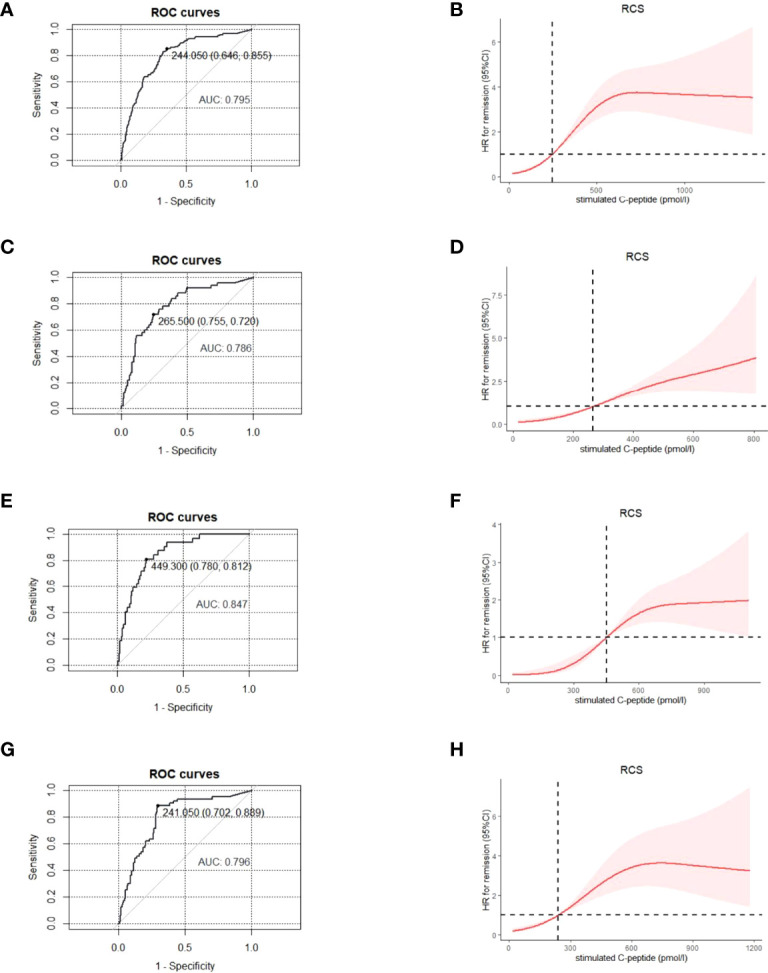
For patients of different age- groups, ROC and RCS curves were used to find the optimal cut-off point for stimulated C-peptide to define the partial remission, with GA≤ 23% and insulin dose ≤0.5 u/kg/day being the diagnostic criteria. All age- groups: ROC **(A)**, RCS **(B)**; ≤ 12 years group, ROC **(C)**, RCS **(D)**; 12-18 years group, ROC **(E)**, RCS **(F)**; ≥ 18 years group, ROC **(G)**, RCS **(H)**. ROC, receiver operating characteristic; RCS, restricted cubic spline; GA, glycated albumin.

The average stimulated C-peptide level was much higher in patients during PR [531.8(95%CI: 482.1, 581.4) pmol/L] than that in patients who were not in PR [252.5(95%CI: 230.7, 274.4) pmol/L] (p < 0.001). ROC and RCS curves showed that the corresponding cut-off level for stimulated C-peptide was 244.1 pmol/L (AUC: 0.8) (**Figures 3A** **, B**) for PR diagnosis.

Due to the age-related heterogeneity of clinical features in T1D patients, we explored whether the cut-off value for stimulated C-peptide applied to different age-groups (≤12y, 12-18y and ≥18y). With GA ≤ 23.0% and insulin dose ≤ 0.5u/kg/day being the diagnostic criteria, the optimal cut-off point for stimulated C-peptide was 265.5, 449.3 and 241.1 pmol/L for the ≤12y, 12-18y and ≥18y age-group, respectively (**Figure 3**).

### Consistency Between Different PR Definitions

**Figure 4:**
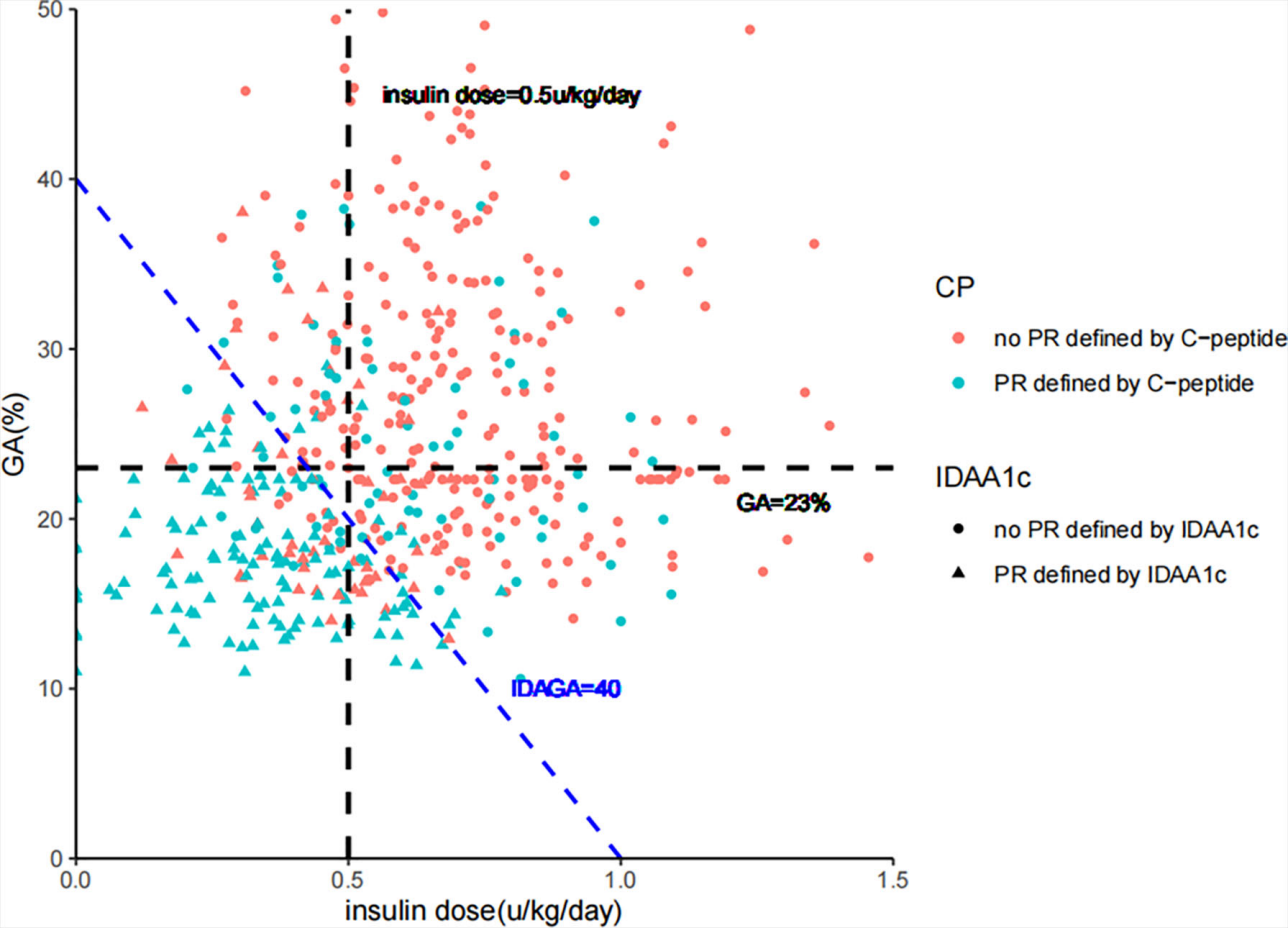
The consistency between diagnosis of partial remission defined by IDAGA≤ 40; IDAA1C ≤ 9; GA≤ 23%/insulin dose≤ 0.5 u/kg/day; and stimulated C-peptide≥265 pmol/L, 450 pmol/L, or 240 pmol/L for the ≤12 years, 12-18 years and ≥18 years group, respectively. GA, glycated albumin; IDAGA, insulin dose-adjusted glycated albumin; IDAA1C, insulin dose–adjusted A1C.

High consistency could be observed between different PR definitions (stimulated C-peptide, GA/insulin dose, IDAGA, and IDAA1C), with GA/insulin dose and IDAGA showing the highest consistency, as expected (**Figure 4**). However, the ratio for diagnostic inconsistency between stimulated C-peptide and GA/insulin dose was 24.3%. Among the 110 cases who met the criteria of PR by stimulated C-peptide, 44 individuals (40%) did not fulfill the criteria by GA/insulin dose or IDAGA. Furthermore, the clinical characteristics between these patients were compared. And the results showed that individuals who met the PR definition only by stimulated C-peptide but not IDAGA or GA/insulin requirement tended to have higher level of LDL-C, total cholesterol and lower level of eGDR, indicating the existence of dyslipidemia and higher extent of insulin resistance in these patients (**Table 2**).

**Table 2:** The differences in clinical characteristics between patients who met the criteria of PR only by stimulated C-peptide and those by both stimulated C-peptide and GA/insulin requirement or IDAGA.

	patients who met the criteria of PR by stimulated C-peptide but not GA/insulin or IDAGA (n = 44)	patients who met the criteria of PR by both stimulated C-peptide and GA/insulin or IDAGA (n = 66)	p-value
Age (years)	13.3 (10.0-19.8)	16.2 (10.5-30.1)	0.178
Duration of diabetes (months)	2.1 (1.0-4.0)	4.4 (2.6-6.5)	<0.001
waist-hip ratio (%)	0.8 (0.8-0.9)	0.8 (0.8-0.9)	0.997
Hemoglobin A1c (%)	9.1 (6.8-12.3)	6.5 (5.8-7.2)	<0.001
Systolic blood pressure (mmHg)	105.3 ± 10.6	109.5 ± 14.7	0.150
Diastolic blood pressure (mmHg)	67.5 (59.3-71.8)	64.0 (57.0-70.0)	0.372
HDL-C (mmol/l)	1.6 ± 0.4	1.5 ± 0.4	0.253
LDL-C (mmol/l)	2.3 (1.8-3.0)	1.9 (1.6-2.3)	0.011
Total Cholesterol (mmol/l)	4.3 (3.6-5.2)	3.7 (3.2-4.3)	0.009
Triglyceride (mmol/l)	0.7 (0.5-1.0)	0.6 (0.4-0.8)	0.067
eGDR (mg/kg/min)	8.8 (7.1-10.0)	10.4 (9.7-11.4)	<0.001

Data are presented as the mean ± standard deviation (SD) or median (25th-75th percentile). HDL-C, high density lipoprotein cholesterol; LDL-C, low density lipoprotein cholesterol; eGDR, estimated glucose disposal rate.

## Discussion

We proposed a new definition of PR in T1D using GA and insulin dose, and transformed the definition into a quantitative measure, namely IDAGA. In addition, our study demonstrated that in different age-groups (≤12y, 12-18y and ≥18y), the cut-off value to diagnose PR was different for stimulated C-peptide, but was relatively consistent for IDAGA. To our knowledge this is the first study to define PR with GA/insulin dose, and come up with the age-dependent cut-off values of stimulated C-peptide for PR diagnosis.

As a special stage in T1D, the accurate diagnosis of PR is of particular clinical and research importance. For patients with hyperglycemia onset at older ages, such improved glycemic control should be attached importance to avoid the misdiagnosis of T1D as type 2 diabetes (T2D) (15). Over half of the newly onset T1D cases occur in adults (16), more than half of whom will enter PR (11). Without the right classification, these patients will not receive optimal care and management restricted to those with T1D, such as carbohydrate counting, continuous glucose monitoring and insulin-pump therapy (1, 17, 18). And timely PR diagnosis facilitates adjustments of the therapy, so as to prevent acute events during the first year of T1D (19). Though PR only lasts for no more than a year, the occurrence of PR can also help with the prediction of long-term diabetes complications in T1D patients (3–5, 20).

Various PR definitions have been proposed, but there is no consensus on an easily usable measurement for patients of all ages (2). In our study, GA/insulin requirement was used to define PR. Compared with hemoglobin, albumin is approximately 10 times more sensitive to glycation, and has a shorter half-life, making GA a better non-fasting marker for glycemic fluctuation (21). Several research has proposed the GA cut-off values to be used in the diagnosis of diabetes, but no unified conclusion has been drawn (22–25). According to our linear regression model, the GA concentration corresponding to HbA1c=7.5% was around 23.0%, similar to the values from previous research (ranging from 20.9% to 24.0%) (26–28), and the slight difference might result from the status of diabetes and ethnic groups of the subjects enrolled.

Due to the age-related heterogeneity in T1D, the cut-off points of IDAGA and stimulated C-peptide were investigated in different age-groups in our study. Since T1D has long been considered a childhood-onset disease, data of T1D in other age-groups are limited, and so does the research related to PR (10, 11, 29). The most commonly used PR definition, IDAA1C, was also derived from subjects aged under 16 years old (30). However, adults have been reported to comprise more than half of the newly diagnosed T1D cases (16), and PR has also been proved to demonstrate different patterns in children, teenagers and adults (11). In addition, the relationship between C-peptide and HbA1c or insulin use varies among different age-groups (31). Therefore, subjects in our study were divided into three age-groups to verify the impact of age on the measurements to diagnose PR. As expected, with fixed GA and insulin dose, stimulated C-peptide showed age-dependent thresholds for PR diagnosis, which emphasizes the consideration of age when diagnosing PR. And the highest cut-off point occurred in the 12-18y patients for stimulated C-peptide, which reflected the effect of puberty on metabolism. In T1D, puberty is a period of dynamic physiological changes that has emerged as a challenge in pediatric care (32), and T1D adolescents exhibit the worst glycemic control compared to children and adults (33). Hormonal changes and insulin resistance (34), treatment adherence (35), and family dynamics could possibly influence the metabolic control during puberty (36).

PR is characterized by the transient recovery of β-cell function and improved glycemic control, and measurements reflecting these two aspects have been be used to define PR (2). In our study, the consistency between the different measurements (stimulated C-peptide, GA/insulin dose, and IDAGA) was investigated. Although high consistency could be seen, about 40% of patients only met the PR definition by stimulated C-peptide, but not IDAGA or GA/insulin dose. And this is consistent with a recent finding that more than 55% of the participants with C-peptide levels > 300 pmol/L were not in PR defined by the IDAA1C, who showed lower level of insulin sensitivity (37). For our patients with high C-peptide level but unsatisfying glucose control, higher level of insulin resistance was also observed, which should be emphasized in clinic. Insulin resistance refers to a weak biological response to certain level of serum insulin, leading to the requirement of more insulin dosage to achieve the same effect (38). This phenomenon would occur before the onset of T1D and persist afterwards (39), and was estimated by eGDR in our study. The eGDR is a valid surrogate index of clamp-derived measure of insulin resistance in T1D, and has been practiced in both children and adults (40). Major causes of insulin resistance in T1D include insufficient endogenous insulin secretion and the toxic impact of chronic hyperglycemia (39). In addition to higher extent of insulin resistance, dyslipidemia existed in these patients. Thus, for newly diagnosed T1D patients who have high β-cell function but rather poor glycemic control, insulin resistance and dyslipidemia may exist, and combined application of different diagnostic criteria is recommended for the PR diagnosis. Also, early intervention should be taken in order to improve their long-term glycemic control and reduce the risk of developing complications.

Although our results presented a feasible new definition of PR for clinical practice, there are some limitations in our study. First, due to the rather long interval between follow-ups, it was not possible to verify the diagnostic sensitivity with regard of the PR entrance and ending. Second, although age has been shown to correlate with metabolic control in T1D, our definition of PR did not include age. But we took age into consideration during subgroup analysis, and provided the different cut-off values when diagnosing PR in different age-groups. Lastly, we performed the study at a single center with limited number of patients, which may impact its generalizability to other geographical areas or population. Further work is needed to determine whether this definition applies in other racial/ethnic groups.

In conclusion, our study proposed a new definition of the PR using GA and insulin dose. The quantitative form of the definition, namely IDAGA ≤ 40, applied to all age-groups. And the study also, for the first time to our knowledge, provided the age-dependent cut-off points of stimulated C-peptide level for PR diagnosis. In addition, it is not recommended to use stimulated C-peptide alone to diagnose PR for patients with signs of insulin resistance.

## Data Availability Statement

The data analyzed during the current study are available from the corresponding author on reasonable request.

## Ethics Statement

The studies involving human participants were reviewed and approved by Medical Ethics Committee of the Second Xiangya Hospital of Central South University. Written informed consent to participate in this study was provided by the participants’ legal guardian/next of kin.

## Author Contributions

All authors agreed to submit and publish the manuscript. All authors collected and analyzed data. MS and XJ wrote the first draft of the manuscript. XL designed the study and revised the manuscript. All authors had full access to the data, made final decisions about content, vouch for the accuracy and completeness of the analyses and approved the final version for submission. XL had full access to all the data in the study and take responsibility for the integrity of the data and the accuracy of the data analysis.

## Funding

This study was supported by The science and technology innovation Program of Hunan Province (2020RC4044) and Natural Science Foundation of China(8207034059).

## Conflict of Interest

The authors declare that the research was conducted in the absence of any commercial or financial relationships that could be construed as a potential conflict of interest.

## Publisher’s Note

All claims expressed in this article are solely those of the authors and do not necessarily represent those of their affiliated organizations, or those of the publisher, the editors and the reviewers. Any product that may be evaluated in this article, or claim that may be made by its manufacturer, is not guaranteed or endorsed by the publisher.
